# High genomic diversity and candidate genes under selection associated with range expansion in eastern coyote (*Canis latrans*) populations

**DOI:** 10.1002/ece3.4688

**Published:** 2018-12-04

**Authors:** Elizabeth Heppenheimer, Kristin E. Brzeski, Joseph W. Hinton, Brent R. Patterson, Linda Y. Rutledge, Alexandra L. DeCandia, Tyler Wheeldon, Steven R. Fain, Paul A. Hohenlohe, Roland Kays, Bradley N. White, Michael J. Chamberlain, Bridgett M. vonHoldt

**Affiliations:** ^1^ Department of Ecology & Evolutionary Biology Princeton University Princeton New Jersey; ^2^ School of Forest Resources and Environmental Science Michigan Technological University Houghton Michigan; ^3^ Warnell School of Forestry and Natural Resources University of Georgia Athens Georgia; ^4^ Ontario Ministry of Natural Resources and Forestry Peterborough Ontario Canada; ^5^ Trent University Peterborough Ontario; ^6^ USFWS National Forensics Laboratory Ashland Oregon; ^7^ Department of Biological Sciences, Institute for Bioinformatics and Evolutionary Studies University of Idaho Moscow Idaho; ^8^ Department of Forestry and Environmental Resources North Carolina State University Raleigh North Carolina; ^9^ North Carolina Museum of Natural Sciences Raleigh North Carolina

**Keywords:** colonization, dispersal behavior, outlier SNPs, RADseq, range expansion

## Abstract

Range expansion is a widespread biological process, with well‐described theoretical expectations associated with the colonization of novel ranges. However, comparatively few empirical studies address the genomic outcomes accompanying the genome‐wide consequences associated with the range expansion process, particularly in recent or ongoing expansions. Here, we assess two recent and distinct eastward expansion fronts of a highly mobile carnivore, the coyote (*Canis latrans*), to investigate patterns of genomic diversity and identify variants that may have been under selection during range expansion. Using a restriction‐associated DNA sequencing (RADseq), we genotyped 394 coyotes at 22,935 SNPs and found that overall population structure corresponded to their 19th century historical range and two distinct populations that expanded during the 20th century. Counter to theoretical expectations for populations to bottleneck during range expansions, we observed minimal evidence for decreased genomic diversity across coyotes sampled along either expansion front, which is likely due to hybridization with other *Canis* species. Furthermore, we identified 12 SNPs, located either within genes or putative regulatory regions, that were consistently associated with range expansion. Of these 12 genes, three (*CACNA1C*,* ALK*, and *EPHA6*) have putative functions related to dispersal, including habituation to novel environments and spatial learning, consistent with the expectations for traits under selection during range expansion. Although coyote colonization of eastern North America is well‐publicized, this study provides novel insights by identifying genes associated with dispersal capabilities in coyotes on the two eastern expansion fronts.

## 1 INTRODUCTION

Range expansions are a ubiquitous aspect of natural history (Excoffier, Foll, & Petit, 2009). Recent or ongoing range expansions have been documented in a variety of diverse taxa, including butterflies (Braschler & Hill, 2007; Hill et al., 2001; Pateman, Hill, Roy, Fox, & Thomas, 2012), mammals (Balestrieri et al., 2010; Taulman & Robbins, 1996), and birds (Livezey, 2009; Swaegers et al., 2015), as well as numerous plant species (Ariani, Mier, & y Teran, J., & Gepts, P., 2017; Colautti & Barrett, 2013; Voss, Eckstein, & Durka, 2012). Yet, despite the widespread prevalence of substantial range expansions, comparatively few empirical studies have explored the genetic or genomic consequences of recent or ongoing expansions, with some exceptions (Hagen, Kopatz, Aspi, Kojola, & Eiken, 2015; Norén et al., 2015; Heppenheimer et al., 2018).

Broadly, range expansion is expected to result in reduced genome‐wide diversity relative to the core range as a consequence of small population sizes and serial founder events (Mayr, 1954; Nei, Maruyama, & Chakraborty, 1975). Strong population structure is also expected along the expansion axis, with recently expanded populations often representing differentiated genetic clusters from those in the core range (Ibrahim and Nichols, 1996). Though demographic factors, such as fecundity and population density, influence population structure and genome‐wide diversity of recently expanded populations (Hagen et al., 2015), natural selection of traits (i.e., reproduction, dispersal) associated with range expansions may also play an important role. Theoretically, traits facilitating range expansion should experience differential selection pressures along the axis of expansion (Travis & Dytham, 2002; Phillips et al., 2008; Burton, Phillips, & Travis, 2010). For instance, genes associated with exploratory behavior and dispersal abilities are predicted to be beneficial at the front of the expansion axis given such traits directly facilitate movement into and subsequent colonization of a new habitat (Burton et al., 2010; Hughes, Dytham, & Hill, 2007; Phillips, Brown, Travis, & Shine, 2008; Travis & Dytham, 2002). Reproductive traits are also predicted to be under selection, as reduced competition and smaller population sizes at the front of the expansion may favor increased reproductive effort (Burton et al., 2010).

Although predictions for adaptive evolution during range expansion are well described in theory, it is a challenge in practice to identify loci under selection at the range periphery for several reasons. First, a stochastic phenomenon known as “allele surfing”, a consequence of serial founder events and drift at the expansion front, may drive even deleterious alleles to high frequencies along the expansion axis. This process has a strong theoretical basis (Edmonds, Lillie, & Cavalli‐Sforza, 2004; Klopfstein, Currat, & Excoffier, 2006) and been suggested in empirical studies for a range of taxa (Hofer, Ray, Wegmann, & Excoffier, 2009; Gralka et al., 2016; Streicher et al., 2016). Therefore, identifying genomic variants with substantial changes in frequency along the expansion axis alone is not a sufficient evidence of recent selection. Additionally, variation in allele frequency may be driven by environmental factors (e.g., novel habitats and food resources) that occur in the expanding range but are independent of traits (e.g., dispersal and reproductive capabilities) that directly facilitate range expansion.

While the effects of allele surfing and environmental factors cannot be completely accounted for when studying range expansion, replicate expansion fronts across distinct environments can help disentangle the relative impact of these forces (Swaegers et al., 2015; White, Perkins, Heckel, & Searle, 2013). As allele surfing is a stochastic process, it is less likely that the same genomic variant would undergo a frequency shift in the same direction relative to the historical range along multiple independent expansion axes. Similarly, when species traverse distinct environments, increases or decreases in frequency at the same loci are less likely to be driven by local adaptation. Therefore, genomic variants that undergo similar frequency shifts across multiple independent axes of expansion are reasonable candidates for range expansion genes.

Coyotes (*Canis latrans*) provide a tractable system to address questions related to range expansion genomics. Confined to the western and central regions of North America prior to 1900 (Nowak, 1979, 2002 ; Young & Jackson, 1951), hereafter referred to as the coyote historical range, coyotes have substantially expanded their geographic range over the last century to occupy every continental US state and Canadian province (Hody & Kays, 2018). Here, we focus on the eastward expansion across the midwestern US and southeastern Canada, culminating along the eastern seaboard. This expansion began in the early 20th century and followed two broad expansion routes across distinct environments. In the northeast, coyotes expanded across the Great Lakes region of the United States and Canada into New England, New York, and Pennsylvania. The southeastern expansion occurred at a slower rate and followed an approximate trajectory through Louisiana, Alabama, and Georgia, with initial reports of coyotes in the Carolinas as recently as the 1980s (DeBow, Webster, & Sumner, 1998). Though fine‐scale variation in expansion routes has been documented for the northeast (Kays, Curtis, & Kirchman, 2010; Wheeldon et al., 2010), a recent genetic survey (Heppenheimer et al., 2018) supports two genetically distinct eastern coyote populations across the eastern seaboard that correspond to these broadly described northeastern and southeastern expansion routes, suggesting that fine‐scale expansion routes have likely converged.

In addition to geographic isolation, each expansion front represents distinct ecoregions in North America. For example, the northeastern expansion front is primarily northern forests and eastern temperate forests, which is further divided primarily into mixed wood shield, Atlantic Highlands, and mixed wood plains (Omernik & Griffith, 2014). In contrast, the southeastern expansion front is almost entirely eastern temperate forests, but transitions to tropical wet forest and great plains designations along the Gulf of Mexico (Omernik & Griffith, 2014). Furthermore, each expansion front also differs in the presence and abundance of closely related *Canis* species. Generally, under a range expansion scenario, hybridization between closely related and previously isolated species may occur as a result of low population density of the expanding species along the range periphery (Seehausen, 2004). As such, coyote hybridization with remnant populations of eastern (*C. lycaon*) and/or gray wolves (*C. lupus*) has been documented along the northeastern expansion route (Kays et al., 2010; Rutledge, Garroway, Loveless, & Patterson, 2010; vonHoldt et al., 2011; vonHoldt, Kays, Pollinger, & Wayne, 2016), as well as with red wolves (*C. rufus*) in the southeastern expansion front (Nowak, 2002). In particular, red wolves are believed to be extirpated outside of the North Carolina recovery area, but hybridization between red wolves and coyotes is well documented within that area (Bohling et al., 2016; Hinton, Gittleman, Manen, & Chamberlain, 2018). Further, several previous studies have also shown that eastern coyote populations have interbred with domestic dogs (Adams, Leonard, & Waits, 2003; Wilson, Rutledge, Wheeldon, Patterson, & White, 2012; Wheeldon, Rutledge, Patterson, White, & Wilson, 2013; Monzõn, Kays, & Dykhuizen, 2014). While there is evidence that these hybridization events have been adaptive (vonHoldt et al., 2016), it is important to note that the full genome‐wide consequences and the geographic extent of interspecies hybridization have not been documented throughout the entire eastern range.

Overall, our objectives were to quantify genomic structure and diversity across the historical coyote range and the two recently expanded eastern coyote populations. We then identify outlier loci that may have been under selection in both populations as a result of range expansion. We predict that population structure will correspond to the known demographic history of North American coyotes, that is, a historical range population and two distinct recently expanded groups. In accordance with theoretical assumptions, we expect reduced genomic diversity in the two recently expanded eastern populations relative to the historical range. However, hybridization with other *Canis* species may result in deviations from this expectation. Finally, we expect genomic variants that underwent frequency shifts in the same direction in both groups to have putative functions related to range expansion, such as dispersal and reproduction.

## 2 METHODS

### 2.1 Sample collection

**Figure 1:**
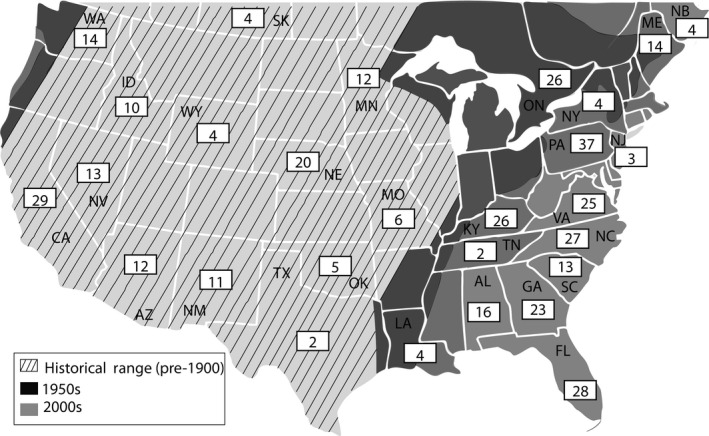
Map of coyote historical range, 1950s range, 2000s range, and sample size per location. Ranges are approximate and modified from Hody and Kays (2018)

We obtained coyote blood and tissue (e.g., liver, kidney, tongue) from state management programs (Princeton IACUC #1961A‐13), government organization archives (e.g., Florida Fish and Wildlife, US Department of Agriculture, Ontario Ministry of Natural Resources and Forestry), or museum archives (New York State Museum, Oklahoma Museum of Natural History). In all cases, state or province of origin was documented (Figure 1), and in many cases, sex, approximate age, and fine‐scale geographic data were also known. Samples were collected between 1998 and 2017, with the majority collected within the last 10 years (2008–2017). Samples with unknown collection dates were either known or assumed to fall within the approximate time period (Supporting information Table S1).

For downstream analyses, we considered samples collected from AZ, CA, ID, MN, MO, NE, NM, NV, OK, SK, TX, WA, and WY, to be part of the historical range (i.e., pre‐1900; Figure 1) as described by Hody and Kays (2018). Additionally, samples collected from ME, NB, NJ, ON, and PA were considered part of the northeast expansion, and samples collected from AL, FL, GA, KY, LA, NC, SC, TN, and VA were considered part of the southeastern expansion.

### 2.2 Sampling and DNA extraction

High molecular weight genomic DNA was extracted from all samples with either the Qiagen DNeasy Blood and Tissue Kit or the BioSprint 96 DNA Blood Kit in conjunction with a Thermo Scientific KingFisher Flex Purification platform, in both cases following instructions provided by the manufacturer. We quantified DNA concentration with either PicoGreen or Qubit 2.0 fluorometry and standardized samples to 5 ng/μl. Only high‐quality DNA samples, as determined by the presence of a high molecular weight band on a 1% agarose gel, were retained for sequencing.

### 2.3 RADsequencing and bioinformatics processing

We prepared genomic libraries following a modified restriction‐associated DNA sequencing (RADseq) protocol described by Ali et al. (2016). Briefly, samples were digested with *sbfI,* and a unique 8 bp barcoded biotinylated adapter was ligated to the resulting fragments. Samples were then pooled (96–153 samples/pool) and randomly sheared to 400 bp in a Covaris LE220. Following shearing, we used a Dynabeads M‐280 streptavidin bead binding assay to enrich for adapter‐ligated fragments. Final sequencing libraries were then prepared using either the NEBNext Ultra DNA Library Prep Kit or the NEBNext UltraII DNA Library Prep Kit. Size selection was made for 300–400 bp insert with Agencourt AMPure XP magnetic beads, which were also used for library purification. Libraries were standardized to 10 nM and sequenced (2X150nt) on two lanes on the Illumina HiSeq 2500.

As this RADseq protocol is unique in that the barcode may be on either the forward or reverse read, data processing was required prior to variant calling. Accordingly, forward and reverse raw sequencing reads were processed such that any read containing the remnant *sbfI* cut site and one of the possible barcodes were aligned in a single file, while the matching read pairs that lacked the cut site were aligned in a separate file, and all remaining reads were discarded. This was accomplished using a custom Perl script (flip_trim_sbfI_170601.pl, see Supporting information).

Additional data processing was then conducted in STACKS v 1.42 (Catchen, Hohenlohe, Bassham, Amores, & Cresko, 2013). First, reads were demultiplexed using *process_radtags*, allowing a 2 bp mismatch for barcode rescue and discarding reads with either uncalled bases or a low‐quality score (<10) within a sliding window of 0.15. Next, PCR duplicates were removed with the paired‐end sequencing filtering option in *clone*_*filter*. We excluded all samples with low read count (<500,000) after removal of PCR duplicates from further analysis. Remaining samples were then mapped to the dog genome CanFam3.1 assembly (Lindblad‐Toh et al., 2005) in Stampy v 1.0.21 (Lunter & Goodson, 2011). To reduces biases associated with incorrectly mapped loci (e.g., paralogs), we additionally filtered mapped reads for a minimum MAPQ of 96 and converted to bam format in Samtools v 0.1.18 (Li et al., 2009).

We completed SNP calling in STACKS following the recommended pipeline for reference mapped data (i.e., *pstacks → cstacks → sstacks → populations;* Catchen et al., 2013). In *pstacks*, we required a minimum depth of coverage of three to report a stack (‐m 3). *Cstacks* and *sstacks* were run as recommended by Catchen et al. (2013). The *populations* module was run twice to optimize final sample selection to reduce both missing data and biases resulting from uneven sampling across locations. First, we allowed only the first SNP per locus (‐‐*write_single_snp*) to be reported but did not apply any missing data thresholds. We then evaluated the per‐individual genotyping success, as measured by missingness per individual in Plink v 1.90b3i (Purcell et al., 2007), and removed individuals with a high level of missing data (>80% missing). We also removed samples from locations where *n* = 1. In our second run of *populations*, we included only this reduced set of samples, required that reported loci be genotyped in 90% of individuals (−*r* = 0.9), and again restricted analysis to only the first SNP per locus. Only this latter dataset was used for subsequent analyses. Following SNP calling, we filtered for statistical linkage disequilibrium in Plink with the argument *‐‐indep‐pairwise* 50 5 0.5.

All SNPs were annotated as genic (intron or exon), within a promoter (i.e., within 2 Kb of transcription start site following vonHoldt, Heppenheimer, Petrenko, Croonquist, and Rutledge (2017)), or intergenic using an in‐house python script (chr_site.py; See supporting information). All intergenic SNPs were compiled in a second genotype dataset and filtered for Hardy–Weinberg Equilibrium (HWE) in Plink with the argument—hwe 0.001. These intergenic, HWE‐filtered genotypes were presumed neutral in downstream analyses (hereafter, putatively neutral loci). Additionally, we compiled a third dataset of putatively functional loci consisting of all SNPs annotated as genic or within 2 Kb of a transcription start site (hereafter, genic loci).

### 2.4 Population structure analysis

To visualize clustering in our data and identify strong outliers, we conducted a Principal Component Analysis using our full SNP dataset with *flashPCA* (Abraham & Inouye, 2014). We identified one strong outlier originating from Ontario, which may be a misidentified eastern or gray wolf (*C. lycaon* or *C. lupus*). This individual was removed from further analyses.

Following the removal of strong outliers (e.g., putatively misidentified wolves), we determined the most likely number of genomic clusters represented by the data, by conducting an analysis of population structure in ADMIXTURE v1.3. (Alexander, Novembre, & Lange, 2009) with the cross‐validation flag. We evaluated *K* = 1–10, with the *K* value with the lowest cross‐validation (cv) score indicative of the best fit K. ADMIXTURE is similar in principle to the classic Bayesian software STRUCTURE (Pritchard, Stephens, & Donnelly, 2000), but uses a maximum likelihood framework and is more computationally efficient for SNP data.

### 2.5 Genomic diversity

Standard metrics of genomic diversity for each sampling location, including private allele counts as well as observed (*H_o_*) and expected (*H_e_*) heterozygosity, were calculated in STACKS across all loci. To determine whether trends of heterozygosity differed based on which part of the genome was surveyed, we recalculated both *H_o_* and *H_e_* across putatively neutral loci and genic loci. Allelic richness (*A_r_*) and private allelic richness (*A*_pr_) were calculated using a rarefaction approach implemented in ADZE v 1.0 (Szpiech, Jakobsson, & Rosenberg, 2008), where the maximum standardized sample size was set to the smallest n for the samples considered (i.e., 176 when comparing the historical range, northeast expansion, and southeast expansion).

Pairwise *F*_ST_ values between all sampling locations over all loci were calculated in STACKS and we tested for isolation by distance (IBD) within the coyote historical range, as well as within each recently expanded eastern population with a series of Mantel tests implemented in *ade4* v1.7‐11 (Dray & Dufour, 2007) in R v3.3 (R Core Team, 2013). Pairwise *F*_ST_ were linearized following Rousset (1997), geographic distances were calculated as the shortest straight‐line distance between sampling locations, and significance was assessed from 9,999 permutations.

### 2.6 Identification of loci associated with range expansion

To identify loci as candidates for selection during range expansion, we restricted analyses to individuals with high cluster assignments as identified in the ADMIXTURE analysis (*Q* ≥ 0.8). Additionally, to prevent biases resulting from long‐distance dispersers, we removed three individuals that had high cluster assignments to different populations from which they were sampled (e.g., sampled from Louisiana but clustered with the northeast group). Though restricting the analyses to individuals with high cluster assignments may inflate the genetic distinction between the historical range and the recently expanded coyote populations, we believe the inferred historical range population to be the best available representation of pre‐range expansion allele frequencies, as there is likely ongoing contemporary gene flow between coyotes in the historical range and both recently expanded populations.

As *F*_ST_ outlier‐type approaches to identify loci under selection are prone to high rates of false positives, especially among populations with complex demographic histories, we used two distinct approaches to identify loci putatively under selection. First, a Bayesian framework to detect outlier loci was implemented in BAYENV2 (Coop, Witonsky, Rienzo, & Pritchard, 2010; Günther & Coop, 2013). This method accounts for evolutionary nonindependence between populations by first calculating covariance in allele frequencies at a set of putatively neutral loci. Candidate functional SNPs are then evaluated one at a time under a model that assumes a linear relationship between an environmental variable and allele frequency compared to a model given by the neutral covariance matrix and a corresponding Bayes factor is calculated. This method has been suggested to outperform other *F*_ST_ outlier‐like methods (e.g., FDIST2, BayeScan) in the case of range expansion (Lotterhos & Whitlock, 2014). In the BAYENV2 analysis, the environmental variable of interest was the linear distance from the coyote historical range (e.g., White et al., 2013). To avoid biases induced by allele frequencies at any one sampling location within the historical range, all states or provinces within the historical coyote range were treated as a single sampling location. Distances for sampling locations outside of the historical range were calculated as the shortest straight‐line distance from the approximate midpoint of the sampled regions within the historical range. The northern and southern expansion fronts were analyzed separately. In both cases, the putatively neutral loci used to generate the control covariance matrix were intergenic SNPs in HWE, filtered further to remove any monomorphic loci between the populations compared. Similarly, the candidate SNPs were all SNPs annotated as genic (intron or exon) or within 2 Kb of a transcription start site, again filtered to remove monomorphic loci between populations. Genotype files for both SNP datasets were converted to BAYENV2 format in PGDSpider v 2.1.13 (Lischer & Excoffier, 2012). For each expansion front (historical to northeast & historical to southeast), loci were ranked by Bayes factor and the top 3% of SNPs were retained for further analysis. Loci were considered candidate genes under selection during range expansion if the same SNP occurred on both lists.

Second, we used a principal component‐based approach implemented with the R package PCadapt (Luu, Bazin, & Blum, 2017). This method first performs a centered, scaled principal component analysis on genome‐wide SNPs and then identifies significant outliers with respect to population structure given by the first *K* principal components. Specifically, PCadapt identifies outliers based on the Mahalanobis distance, which describes multidimensional distance of a point from the mean. Simulations indicate that PCadapt is less prone to type II error than alternative methods (e.g., BayeScan) and PCadapt is expected to perform well under a variety of complex demographic scenarios, including range expansion (Luu et al., 2017).

**Figure 2:**
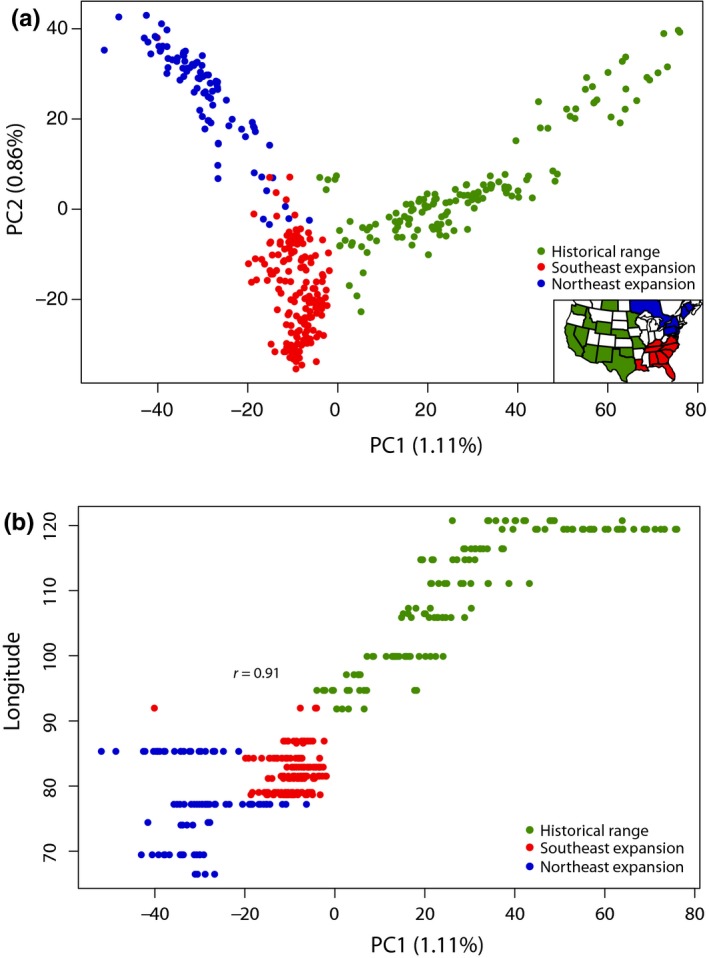
(a) Principal component analysis (PCA) of molecular data for all 394 coyotes. Insert: Geographic distribution of sampling locations. (b) Correlation between PC1 and longitude of sampling location (Pearson's *r* = 0.91: *p* < 2.2 × 10^−16^)

In the PCadapt analysis, the northeastern and southeastern expansion fronts were analyzed separately. In both cases, we identified outliers with respect to underlying population structure given by PC1. We chose to retain only PC1, rather than selecting the optimal number of PCs based on conventional methods (e.g., scree plot), as PC1 primarily captured the major axis of range expansion and therefore corresponded to the level of population structure relevant for this study (See Figure 2). To evaluate significance, *p*‐values for each SNP were transformed into q‐values and SNPs with *q*‐values <0.05 were retained, therefore controlling for a false discovery rate (FDR) of 5%. This was implemented in the R package qvalue v2.6 (Bass, Dabney, & Robinson, 2018). Again, we only considered SNPs that were significant in both historical and northeast and historical and southeast comparisons as candidates for loci under selection during range expansion.

Gene functions and gene ontology biological process annotations of all outlier SNPs identified by both the analyses methods were inferred using Ensembl (release 91; Zerbino et al., 2017) and AmiGO 2 accessed in February 2018 (Carbon et al., 2009). We conducted a gene ontology (GO) biological process overrepresentation enrichment analysis on outlier sites located in functional genomic regions (i.e., intron, exon, or promoter) using WebGestalt (Zhang, Kirov, & Snoddy, 2005; Wang et al., 2013). We used our genic SNP dataset as the reference set for the enrichment analysis, and significance was evaluated using an FDR threshold of 5%. Additionally, we used the Ensembl Variant Effect Predictor (McLaren et al., 2016) to predict the functional effect of all outlier SNPs.

## 3 RESULTS

### 3.1 Genotyping

Among the final samples retained for analyses, raw sequencing read counts per sample ranged between 770,960 and 13,299,663, with an average of 2,466,167 reads. Filtering for PCR duplicates prior to SNP calling removed between 5.32% and 56.15% (average: 21.48%; Supporting information Table S2) of reads, leaving between 582,946 and 10,786,513 (average: 1,908,634; Supporting information Table S2) reads assigned to each unique barcode. Mappability to the dog genome following the removal of reads with low MAPQ scores ranged from 63.77% to 79.20% (average: 74.04%).

We sequenced a total of 3,597,305 restriction‐associated sites (RADtags), 24,139 of which contained at least one variable site in 394 coyotes. Following LD filtering, our full dataset consisted of 22,935 biallelic SNP loci, with a total genotyping rate of 93.3% (Supporting information Figure S1). Average per‐individual missingness was highest in the northeast (mean missing = 10.5%) and slightly lower in both the southeast (6.0%) and the historical range (5.4%; Supporting information Figure S2). Average depth of coverage across all individuals was 11.333 with a standard deviation of 6.626 and was similar among the three regions surveyed (historical range: 11.420, stdev = 5.647; northeast: 11.634, stdev = 9.991; southeast: 11.096, stdev = 4.988; Supporting information Figure S3). Furthermore, overall allele balance for all heterozygotes (i.e., minor allele coverage relative to total site coverage) was 0.496 (stdev = 0.113) and again similar across all three regions (historical range: 0.496, stdev = 0.113; northeast: 0.493, stdev = 0.114; southeast: 0.497, stdev = 0.112).

Overall, we primarily captured rare variation, with an average global minor allele frequency of 1.73%. Further, within each of the three regions sampled, minor allele frequencies were typically ≤5% (Supporting information Figure S4). Approximately, half of the sites were intergenic (*n*_intergenic_ = 12,676), with 14,108 SNPs found within genes (*n*_intron_ = 12,024; *n*_exon_ = 1,532; *n*_promoter_ = 552). These annotations sum to >22,935 as SNPs may have multiple annotations (e.g., promoter and intron). Additionally, our putatively neutral dataset, which consisted of intergenic SNPs in HWE, retained 11,518 SNPs. Our genic data included 10,259 SNPs within introns, exons, and promoters.

### 3.2 Population structure corresponds to expansion axis

Our PCA divided sampling locations as predicted, with PC1 (1.11% variance explained) separating samples originating from the historical coyote range from either recently expanded eastern population (Figure 2a). Accordingly, PC1 was significantly correlated with the longitude of sampling location (state or province; Pearson's *r* = 0.91; *p* < 2.2 × 10^−^^16^; Figure 2b). PC2 (0.86% variance explained) primarily separated northeastern sampling locations from southeastern sampling locations. Furthermore, samples from the Mid‐Atlantic contact zone between these two fronts of expansion (North Carolina, Virginia) tended to have intermediate spatial placement on PC2 (Figure 2a).

**Figure 3:**
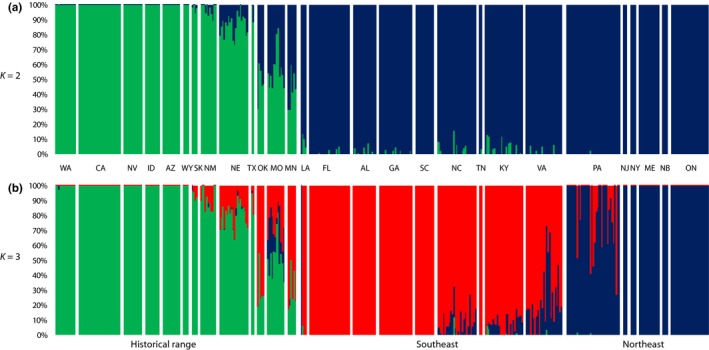
Percent ancestry assignments (Q) at *K* = 2 (a) and *K* = 3 (b) in the admixture analysis

Our ADMIXTURE analysis indicated that three distinct genetic clusters were best represented by the data (cv = 0.107; Figure 3; Supporting information Figure S5). Generally, these clusters were concordant with sampling location, with one cluster corresponding to the historical coyote range, a second cluster corresponding to the northeastern expansion front, and a third cluster corresponding to the southeastern expansion (Figure 3b), that is, samples collected from the historical coyote range showed high assignments to the historical range cluster (Average *Q*_Historical_ = 0.868; Supporting information Table S3), and similarly, samples obtained from either the northeastern or southeastern expansion front were strongly assigned to each respective cluster, average *Q*_Northeast_ = 0.943, and southeast average *Q*_Southeast_ = 0.933 (Supporting information Table S3). We observed moderately high frequencies of intermediate ancestry assignments to both recently expanded eastern population in the Mid‐Atlantic region (e.g., NC, KY, VA, PA; Figure 3b; Supporting information Table S3), consistent with this region as the location of recent secondary contact between the two expansion fronts (Heppenheimer et al., 2018). Despite the clean separation of clusters based on known expansion routes, one sample originating from Louisiana strongly clustered with the northeastern group. Additionally, some sampling locations within the historical range (e.g., MO, OK, NE, MN) exhibited intermediate assignments to the southeastern cluster. Furthermore, clustering at *K* = 2 was consistent with one historical range cluster and one recently expanded group (Figure 3a).

### 3.3 High genomic diversity in recently expanded populations

**Figure 4:**
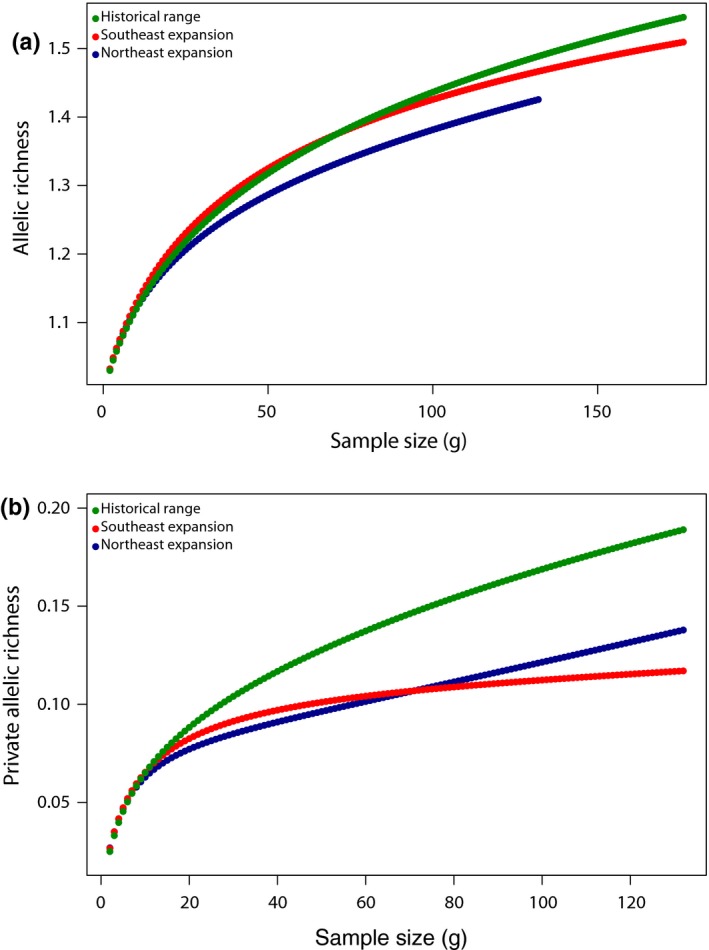
Allelic richness (a) and private allelic richness (b) across all loci as a function of standardized sample size

Genome‐wide heterozygosity was approximately equivalent across the historical range (Average *H_E_* = 0.0264) and northeastern expansion front (Average *H_E_* = 0.0268) and slightly elevated in the southeastern expansion front (Average *H_E_* = 0.0300). These relative trends were similar when analysis was restricted to either putatively neutral loci (Average *H_E_* Historical = 0.0208; Average *H_E_* Northeast = 0.0195; Average *H_E_* Southeast = 0.0235; Supporting information Table S4) or genic SNPs (Average *H_E_* Historical = 0.0256; Average *H_E_* Northeast = 0.0267; Average *H_E_* Southeast = 0.0297; Supporting information Table S4). Furthermore, allelic richness was highest in the historical range and (historical *A_r_* = 1.489, stderr = 0.003; Figure 4a) lower in both expansion fronts (southeast *A_r_* = 1.467, stderr = 0.003; northeast *A_r_* = 1.425, stderr = 0.003; Figure 4a). Private allele counts (Table 1) and private allelic richness (Figure 4b), exhibited a similar trend, with the highest values observed for the historical range (historical *A*_pr_ = 0.189, stderr = 0.002; count = 5,799) and lower values observed in the southeastern front (southeast *A*_pr_ = 0.117, stderr = 0.002; count = 3,578) and northeastern expansion front (northeast *A*_pr_ = 0.138, stderr = 0.002; count = 3,018).

**Table 1:** Summary statistics for all sampling locations (*n* = 394) across 22,935 biallelic SNPs

Sampling locations	*n*	Private alleles	*H_o_*	*H_e_*
Historical Range				
Arizona (AZ)	12	470	0.0254	0.0278
California (CA)	29	895	0.0225	0.0276
Idaho (ID)	10	167	0.0208	0.0237
Minnesota (MN)	12	311	0.0326	0.0324
Missouri (MO)	6	105	0.0199	0.0227
Nebraska (NE)	20	583	0.0250	0.0278
New Mexico (NM)	11	401	0.0276	0.0288
Nevada (NV)	13	406	0.0244	0.0272
Oklahoma (OK)	5	162	0.0296	0.0289
Saskatchewan (SK)	4	132	0.024	0.0244
Texas (TX)	2	146	0.0281	0.0225
Washington (WA)	14	227	0.0260	0.0278
Wyoming (WY)	4	115	0.0214	0.0220
Overall historical range	142	5,799^a^	0.0252	0.0264
Northeast expansion				
Maine (ME)	14	499	0.0231	0.0292
New Brunswick (NB)	4	107	0.0207	0.0228
New Jersey (NJ)	3	47	0.0259	0.0233
New York (NY)	4	65	0.0257	0.0222
Ontario (ON)	26	464	0.0305	0.0324
Pennsylvania (PA)	37	1,511	0.0233	0.0307
Overall Northeast Expansion	88	3,018^a^	0.0249	0.0268
Southeast expansion				
Alabama (AL)	16	136	0.0319	0.0326
Florida (FL)	28	843	0.0262	0.0311
Georgia (GA)	23	151	0.0300	0.0318
Kentucky (KY)	26	280	0.0298	0.0318
Louisiana (LA)	4	125	0.0268	0.0284
North Carolina (NC)	27	406	0.0296	0.0321
South Carolina (SC)	13	100	0.0325	0.0324
Tennessee (TN)	2	32	0.0295	0.0228
Virginia (VA)	25	305	0.0221	0.0270
Overall Southeast Expansion	164	3,578^a^	0.0287	0.0300

Note

*n*, sample size; *H_o_*, observed heterozygosity; *H_e_*, expected heterozygosity.

a Private allele counts per state/province are not expected to sum to the overall private allele count per region, as alleles may be private to a region without being private to any individual state or province.

We found no evidence of isolation by distance in the historical coyote range (Mantel *R* = 0.154, *p* = 0.152), or in either recently expanded eastern population (northeast Mantel *R* = −0.288, 0.715; southeast Mantel *R* = 0.846, *p* = 0.327).

### 3.4 Outlier loci associated with range expansion

**Figure 5:**
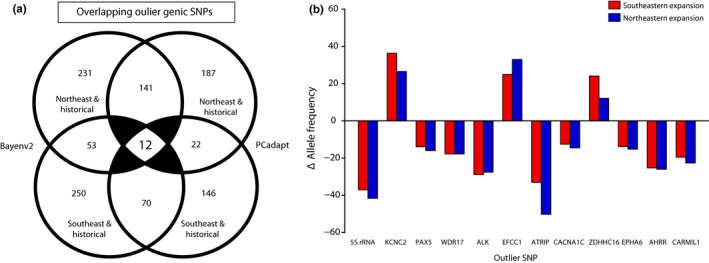
(a) Outlier SNP counts identified by the BAYENV2 and PCadapt analyses. (b) Change in allele frequencies of the outlier SNPs identified in both analyses in the northeast and southeast expansion fronts relative to the historical range

With the BAYENV2 approach, the Bayes factors for the top 3% of ranked SNPs ranged 20.80–53,564 (mean = 3,558.25) for the northeast and historical range analysis and 13.10–130,670,000 (mean 2,713,743.43) for the southeast and historical range analysis (Table 2; Supporting information Table S5). A total of 53 genic SNPs were shared among the top 3% of ranked SNPs in both the northeast expansion and historical range and southeast expansion and historical range analyses (Figure 5a). These SNPs were primarily intronic (*n*_intron_ = 45), with six exonic SNPs, and two located in putative promoter regions (Table 2; Supporting information Table S5). With the PCadapt approach, 59 SNPs were significant outliers (FDR 5%) in both the northeast expansion and historical range and southeast expansion and historical range analyses (Figure 5a). Of these, 22 SNPs were within genes (*n*_intron_ = 20; *n*_promoter_ = 2) and 37 were intergenic (Table 2; Supporting information Table S5). Though these two analyses methods to identify outlier SNPs are similar, BAYENV2 is based on a priori defined groups (i.e., sampling location), while PCadapt is based on principal component scores without predefined groups. However, as PC1 was highly correlated with the expansion axis (see Figure 2b), there was no discordance between placement along PC1 and the categorization of samples as either historical or recently expanded. Accordingly, we consider this analysis to be directly comparable and focus our results and discussion on SNPs and genes identified by both analyses (Lotterhos & Whitlock, 2015).

**Table 2:** Outlier SNPs putatively associated with range expansion identified in both the outlier detection analyses

Chr	Position	Gene	Gene description	Gene Region	BF Northeast	BF Southeast	*p* Northeast	*p* Southeast
2	43,210,227	*5S_rRNA*	5S ribosomal RNA	Prom	53,564	5,477,400	1.3 × 10^−^^5^	9.7 × 10^−^^7^
10	15,795,366	*KCNC2*	Potassium voltage‐gated channel subfamily C member 2	Intron	389.1	130,670,000	1.5 × 10^−^^5^	1.6 × 10^−^^7^
11	53,204,490	*PAX5*	Paired box 5	Intron	253.3	20.9	2.3 × 10^−^^3^	1.8 × 10^−^^5^
16	53,466,454	*WDR17*	WD repeat domain 17	Intron	27.5	5,244	3.5 × 10^−^^3^	2.6 × 10^−^^6^
17	23,407,156	*ALK*	ALK receptor tyrosine kinase	Intron	49.9	1,050,300	1.1 × 10^−^^4^	1.5 × 10^−^^13^
20	3,062,963	*EFCC1*	EF‐hand and coiled‐coil domain containing 1	Intron	26,286	64,928	6.5 × 10^−^^6^	7.2 × 10^−^^5^
20	40,628,561	*ATRIP*	ATR interacting protein	Intron	122.2	474.3	6.6 × 10^−^^7^	1.8 × 10^−^^4^
27	44,159,565	*CACNA1C*	Calcium voltage‐gated channel subunit alpha1 C	Prom	205.4	15	3.4 × 10^−^^3^	1.2 × 10^−^^4^
28	10,746,279	*ZDHHC16*	Zinc finger DHHC‐type containing 16	Intron	178.5	6,046,600	1.8 × 10^−^^3^	4.9 × 10^−^^5^
33	4,379,522	*EPHA6*	EPH receptor A6	Intron	707.3	137.8	1.3 × 10^−^^3^	4.1 × 10^−^^8^
34	11,841,558	*AHRR*	Aryl‐hydrocarbon receptor repressor	Intron	65.3	155,940	3.3 × 10^−^^4^	5.2 × 10^−^^8^
35	23,379,157	*CARMIL1*	Capping protein regulator and myosin 1 linker 1	Intron	232.5	231.6	1.1 × 10^−^^4^	5.9 × 10^−^^7^

Notes

Chr, Chromosome; BF, Bayes Factor; prom, promoter.

Full Biological Process GO annotations for each outlier are given in Supporting information Table S6.

**Figure 6:**
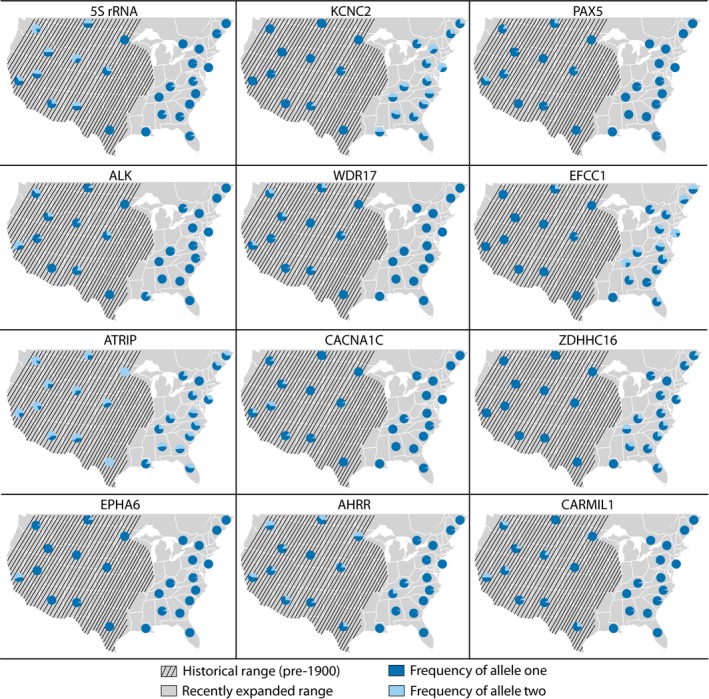
Allele frequencies for all twelve outlier SNPs across sampling locations. Allele one was arbitrarily defined as the most common allele overall (i.e., the major allele)

A total of twelve SNPs of 22,935 were outliers in both the outlier analyses (Table 2; Figure 5a). In all cases, the change in allele frequency relative to the historical range was in the same direction for both recently expanded eastern populations (Figures 5b and 6). For nine of these outlier loci, the less common allele overall (i.e., the global minor allele), decreased in the recently expanded populations, and for the remaining three loci, the minor allele increased in frequency (Figures 5b and 6). In one case, *WDR17*, the minor allele was lost in both of the recently expanded eastern populations (Figure 6). For three additional outlier loci (*PAX5*,* EPHA6* and *CARMIL1*), the minor allele was lost in one of the recently expanded populations and substantially reduced in frequency in the other (Figure 5b). Furthermore, there was only one locus (*ZDHHC16*) where the minor allele was absent from the historical range, but present at appreciable frequencies, in both recently expanded populations (*q*_Northeast_ = 12.12%; *q*_Southeast_ = 24.09%; Figure 6).

In our GO enrichment analysis, these outlier SNPs were not significantly enriched for any biological process (Supporting information Table S6) after applying an FDR threshold of 5%. Furthermore, all sites were annotated as “modifiers” in the VEP analysis, which are defined as variants with no predictable functional effects on coding regions (i.e., noncoding variants).

## 4 DISCUSSION

Recently expanded coyote populations in eastern North America provide a unique opportunity to explore how range expansion shapes genomic diversity at neutral and putatively adaptive loci. Here, we identified three genetic groups of coyotes, which largely correspond to the historical range and two distinct expansion fronts. Instances of discordance between sampling location and cluster assignments were relatively rare, and likely due to recent shared ancestry as well as ongoing gene flow. In particular, coyotes from OK, NE, and MN exhibited intermediate assignments to the southeastern cluster, and coyotes from MO exhibited intermediate assignments to both the southeastern and northeastern clusters. With the exception of MN, this midwestern region has previously been suggested to represent the source population for the southeastern expansion (Nowak, 1979; vonHoldt et al., 2011). Additionally, as coyotes are highly mobile, there is likely ongoing gene flow among the recently expanded populations and those in the historical range, particularly along the eastern extreme. To directly address the relative impacts of shared ancestry and ongoing gene flow in the eastern historical range, a more extensive sampling scheme throughout this region is needed and pre‐range expansion samples from prior to 1900 should also be included.

As in Heppenheimer et al. (2018), we observed comparatively high levels of diversity in both the northeastern and southeastern coyote populations, which is inconsistent with the theoretical (Excoffier et al., 2009) and empirical expectations for recently expanded populations. For example, recent studies reported decreased heterozygosity for populations of bank voles (*Myodoes glareolus*) and damselflies (*Coenagrion scitulum*) in expansion fronts relative to their source populations (White et al., 2013; Swaegers et al., 2015). In contrast, we observed approximately equivalent heterozygosity between coyote populations in the historical and recently established northeastern ranges, and more intriguingly, we observed that coyotes in the southeastern US had slightly greater heterozygosity than those in the historical range. As this trend is consistent across various subsections of the genome (i.e., genic and putatively neutral regions), it is not immediately clear from this study what is driving this lack of reduction in genomic diversity in the recently established eastern populations. However, the observed trends for private allele counts and private allelic richness in coyotes are consistent with a bottleneck scenario, as the historical range was observed to have the highest number of private alleles and the highest private allelic richness. As the loss of rare alleles will have a greater impact on allelic richness than heterozygosity, (Greenbaum et al., 2014), allelic richness has been suggested to be a better indicator of a bottleneck, particularly following range expansion.

The maintenance of relatively high heterozygosity in recently expanded populations could be the result of a selective process, such as balancing selection along the expansion axes. However, it is perhaps more likely that this pattern results from extensive gene flow, either due to long‐distance dispersal by coyotes originating from the historical range or due to interbreeding with other *Canis* species inhabiting the eastern United States or southeastern Canada. While characterizing interspecific hybridization is beyond the scope of the current study, it is likely that these hybridization events have impacted the genetic diversity of eastern coyotes. Future studies should include representative individuals from these potential introgressing species (red wolves, eastern wolves, gray wolves, and dogs) to directly determine the impact of hybridization on the genome‐wide trends of diversity in eastern coyote populations. Interestingly, we note that if hybridization is responsible for the observed heterozygosity trends, our results suggest that interspecific hybridization is most prevalent in the southeastern expansion front, which has received comparatively less attention than coyote/wolf hybridization in the northeastern expansion front, especially on a genome‐wide scale. Accordingly, red wolf/coyote hybridization, particularly outside of the red wolf recovery area in North Carolina (i.e., early range expansion hybridization), is an intriguing area for future research.

Our results, with respect to genomic diversity, are similar to those of vonHoldt et al. (2011), who observed comparable patterns of heterozygosity across similar groups of eastern coyote populations. However, our observed heterozygosity values (Average *H_E_ *= 0.028) are approximately one order of magnitude lower than those reported by vonHoldt et al. (2011) (Average *H_E_* = 0.22). While both estimates are based on genome‐wide SNP data, this discrepancy is likely reflective of the methodological differences of the SNP ascertainment strategies. That is, the canine genotyping array employed in vonHoldt et al. (2011) targeted genomic regions that had been previously screened for diversity, whereas the RADseq methods used in this study are SNP discovery pipeline without a priori information regarding diversity.

Of the twelve SNPs we identified as outliers, several are located within or near genes that have been implicated in phenotypic traits, namely dispersal behaviors, that may be relevant to range expansion. The behavioral consequences of reduced or completely inhibited gene function at three loci (*CACNA1C*,* ALK*, and *EPHA6*) were investigated extensively in a rodent model. For example, mice heterozygous for a *CACNA1C* knockout exhibited reduced locomotion bursts and scanning behavior, as well as increased freezing time, relative to their wild type counterparts (Kabitzke et al., 2017). Additionally, *ALK* knockout mice exhibit enhanced performance in novel object recognition tests, suggesting that this gene plays a role in the ability of animals to explore a novel environment (Bilsland et al., 2008). Finally, *EPHA6* knockout mice demonstrated learning and spatial memory deficits relative to wild type mice. These traits may all be intuitively linked to movement and dispersal capabilities, which are strongly tied to spatial learning (Delgado, Penteriani, Nams, & Campioni, 2009; Saastamoinen et al., 2017).

While these finds are intriguing, the implications for how mutations in these outlier loci impact dispersal capabilities in coyotes should be interpreted with extreme caution. None of these outlier SNPs were found within an exon, and it is unclear from this data whether the genotyped variants directly impact gene expression, or whether these variants are in linkage disequilibrium with one or more nonsynonymous mutations in coding regions. Further, evidence for a dispersal‐related function of these genes is entirely based on mouse studies, and it is unknown if these functions are conserved across mammals. We also did not incorporate any behavioral data for coyotes in this study, and it is unclear if eastern coyotes exhibit differences in exploratory behavior relative to coyotes from the historical range, much less if this behavior is correlated with genotype. Future studies should target the coding sequence of these genes in coyotes to further elucidate how these outlier loci may influence phenotypic traits in expanding coyote populations.

It is additionally possible that our outlier detection approach identified loci that are systematically different between both expansion fronts and the historical range as a result of parallel adaptations to similar environments rather than the range expansion process. While the northern and southern expansion fronts are distinct ecoregions (Omernik & Griffith, 2014), environmental similarities between the regions do exist, most notably in the high abundance of deer. However, diet studies reveal that deer consumption varies widely among eastern coyote populations (Kilgo, Ray, Ruth, & Miller, 2010; Mastro, 2011; Robinson, Diefenbach, Fuller, Hurst, & Rosenberry, 2014) and that deer consumption is also reasonably common throughout the historical range (Ballard, Lutz, Keegan, Carpenter, & deVos Jr, 2001; Carrera et al., 2008; Gese & Grothe, 1995). As such, selection associated with the range expansion process is perhaps more likely than adaptation to deer rich environments, though the possibility remains that outlier SNP frequencies are driven by selection associated with unmeasured environmental variables rather than by range expansion.

One additional outlier locus, *ZDHHC16*, which has putative functions related to eye development, cellular response to DNA damage, heart development, and protein palmitoylation, was monomorphic in the historical population yet polymorphic in both recently expanded populations. There are three general explanations for the origin of this variant in the recently expanded eastern coyote populations: (a) The mutation was present in the historical range, but at an extremely low frequency that was not captured by our sampling, (b) a de novo mutation occurred, either convergently along both expansions fronts, or along one front and then was transferred via intraspecific gene flow, or (c) this allele introgressed from a closely related *Canis* species following a hybridization event and was subsequently transferred via gene flow. As discussed above, interspecies hybridization occurs among *Canis* species, and there is evidence that this has been adaptive in the context of coyote range expansion (Thornton & Murray, 2014; vonHoldt et al., 2016). It is therefore conceivable that *ZDHHC16* represents an additional case of adaptive introgression. However, the data presented here are not sufficient to address questions related to the origin of genomic variants, though this remains an interesting question for future studies regarding the role of hybridization in facilitating range expansion.

Taken together, we present a comprehensive genome‐wide survey of coyote populations across much of the contiguous US as well as southeastern Canada. Despite pronounced geographic structuring among the historical and two recently expanded eastern coyote populations, we did not observe a strong decline in genomic diversity that is characteristic of a range expansion bottleneck, suggesting that coyote range expansion dynamics are more complex than those described in theoretical (Excoffier et al., 2009) and other empirical studies (e.g., White et al., 2013; Swaegers et al., 2015), and is likely attributable to interspecies hybridization. Further, we identify several genomic variants that are candidates for gene regions under selection during range expansion, which provides a critical first step in understanding how functional genomic variation may have facilitated coyote range expansion.

## CONFLICT OF INTEREST

The authors declare no conflict of interest.

## AUTHOR CONTRIBUTIONS

EH, KEB, and BMV designed the study. EH, KEB, ALD, and LYR performed DNA extractions and prepared libraries for sequencing. EH performed analyses and drafted the manuscript. PAH provided technical guidance on laboratory work and bioinformatics. KEB, JWH, BRP, TW, SRF, RK, BNW, and MJC donated samples. All authors contributed to and approved the final manuscript.

## DATA ACCESSIBILITY

RADseq clone‐filtered demultiplexed fastq files and CanFam3.1 mapped bam files have been deposited in the NCBI Sequence Read Archive at accession PRJNA497119 (SAMN10248298‐ SAMN10248691). Genotype calls for 394 coyotes at 22,935 SNPs and associated sample metadata have been deposited to Dryad: https://doi:10.5061/dryad.7f9q2cd.

## References

[ece34688-bib-0001] Abraham, G. , & Inouye, M. (2014). Fast principal component analysis of large‐scale genome‐wide data. PLoS One, 9(4), 1–5. 10.1371/journal.pone.0093766. PMC3981753 24718290

[ece34688-bib-0002] Adams, J. R. , Leonard, J. A. , & Waits, L. P. (2003). Widespread occurrence of a domestic dog mitochondrial DNA haplotype in southeastern US coyotes. Molecular Ecology, 12(2), 541–546. 10.1046/j.1365-294X.2003.01708.x. 12535104

[ece34688-bib-0003] Alexander, D. H. , Novembre, J. , & Lange, K. (2009). Fast model‐based estimation of ancestry in unrelated individuals. Genome Research, 19, 1655–1664. 10.1101/gr.094052.109 19648217 PMC2752134

[ece34688-bib-0004] Ali, O. A. , O’Rourke, S. M. , Amish, S. J. , Meek, M. H. , Luikart, G. , Jeffres, C. , & Miller, M. R. (2016). Rad capture (rapture): Flexible and efficient sequence‐based genotyping. Genetics, 202(2), 389–400. 10.1534/genetics.115.183665. 26715661 PMC4788223

[ece34688-bib-0005] Ariani, A. , Berny Mier y Teran, J. C. , & Gepts, P. (2017). Spatial and temporal scales of range expansion in wild *Phaseolus vulgaris* . Molecular Biology and Evolution, 35(1), 119–131. 10.1093/molbev/msx273. PMC5850745 29069389

[ece34688-bib-0006] Balestrieri, A. , Remonti, L. , Ruiz‐González, A. , Gómez‐Moliner, B. J. , Vergara, M. , & Prigioni, C. (2010). Range expansion of the pine marten (*Martes martes*) in an agricultural landscape matrix (NW Italy). Mammalian Biology, 75(5), 412–419. 10.1016/j.mambio.2009.05.003.

[ece34688-bib-0007] Ballard, W. B. , Lutz, D. , Keegan, T. W. , Carpenter, L. H. , & deVos, J. C. (2001). Deer‐predator relationships: A review of recent North American studies with emphasis on mule and black‐tailed deer. Wildlife Society Bulletin, 29, 99–115.

[ece34688-bib-0008] Bass, A. J. , Dabney, A. , & Robinson, D. (2018). qvalue: Q‐value estimation for false discovery rate control. R package version 2.14.0. Retrieved from http://github.com/jdstorey/qvalue

[ece34688-bib-0009] Bilsland, J. G. , Wheeldon, A. , Mead, A. , Znamenskiy, P. , Almond, S. , Waters, K. A. , … Munoz‐Sanjuan, I. (2008). Behavioral and neurochemical alterations in mice deficient in anaplastic lymphoma kinase suggest therapeutic potential for psychiatric indications. Neuropsychopharmacology, 33(3), 685–700. 10.1038/sj.npp.1301446. 17487225

[ece34688-bib-0010] Bohling, J. H. , Dellinger, J. , McVey, J. M. , Cobb, D. T. , Moorman, C. E. , & Waits, L. P. (2016). Describing a developing hybrid zone between red wolves and coyotes in eastern North Carolina, USA. Evolutionary Applications, 9(6), 791–804. 10.1111/eva.12388. 27330555 PMC4908465

[ece34688-bib-0011] Braschler, B. , & Hill, J. K. (2007). Role of larval host plants in the climate‐driven range expansion of the butterfly *Polygonia c‐album* . Journal of Animal Ecology, 76(3), 415–423. 10.1111/j.1365-2656.2007.01217.x. 17439459

[ece34688-bib-0012] Burton, O. J. , Phillips, B. L. , & Travis, J. M. J. (2010). Trade‐offs and the evolution of life‐histories during range expansion. Ecology Letters, 13(10), 1210–1220. 10.1111/j.1461-0248.2010.01505.x. 20718846

[ece34688-bib-0013] Carbon, S. , Ireland, A. , Mungall, C. J. , Shu, S. Q. , Marshall, B. , & Lewis, S. (2009). AmiGO: Online access to ontology and annotation data. Bioinformatics, 25(2), 288–289. 10.1093/bioinformatics/btn615. 19033274 PMC2639003

[ece34688-bib-0014] Carrera, R. , Ballard, W. , Gipson, P. , Kelly, B. T. , Krausman, P. R. , Wallace, M. C. , … Webster, D. B. (2008). Comparison of Mexican wold and coyote diets in Arizona and New Mexico. Journal of Wildlife Managament, 72(2), 376–381.

[ece34688-bib-0015] Catchen, J. , Hohenlohe, P. A. , Bassham, S. , Amores, A. , & Cresko, W. A. (2013). Stacks: An analysis tool set for population genomics. Molecular Ecology, 22(11), 3124–3140. 10.1111/mec.12354. 23701397 PMC3936987

[ece34688-bib-0016] Colautti, R. I. , & Barrett, S. C. H. (2013). Rapid adaptation to climate facilitates range expansion of an invasive plant. Science, 342(6156), 364–366. 10.1126/science.1242121. 24136968

[ece34688-bib-0017] Coop, G. , Witonsky, D. , Rienzo, A. D. , & Pritchard, J. K. (2010). Using environmental correlations to identify loci underlying local adaptation. Genetics, 185(4), 1411–1423. 10.1534/genetics.110.114819. 20516501 PMC2927766

[ece34688-bib-0018] R Core Team (2013). R: A language and environment for statistical computing. Vienna, Austria: R Foundation for Statistical Computing https://www.R-project.org/.

[ece34688-bib-0019] DeBow, T. , Webster, W. , & Sumner, P. (1998). Range expansion of the coyote, *Canis latrans* (Carnivora: Canidae), into North Carolina; with comments on some management implications. The Journal of the Elisha Mitchell Scientific Society, 114(3), 113–118.

[ece34688-bib-0020] Delgado, M. M. , Penteriani, V. , Nams, V. O. , & Campioni, L. (2009). Changes of movement patterns from early dispersal to settlement. Behavioral Ecology and Sociobiology, 64(1), 35–43. 10.1007/s00265-009-0815-5.

[ece34688-bib-0021] Dray, S. , & Dufour, A. B. (2007). The ade4 Package: Implementing the duality diagram for ecologists. Journal of Statistical Software, 22(4), 1–20. 10.18637/jss.v022.i04

[ece34688-bib-0022] Edmonds, C. A. , Lillie, A. S. , & Cavalli‐Sforza, L. L. (2004). Mutations arising in the wave front of an expanding population. Proceedings of the National Academy of Sciences, 101(4), 975–979. 10.1073/pnas.0308064100. PMC327127 14732681

[ece34688-bib-0023] Excoffier, L. , Foll, M. , & Petit, R. J. (2009). Genetic consequences of range expansions. Annual Review of Ecology, Evolution, and Systematics, 40(1), 481–501. 10.1146/annurev.ecolsys.39.110707.173414.

[ece34688-bib-0024] Gese, E. M. , & Grothe, S. (1995). Analysis of coyote predation on deer and elk during winter in Yellowstone National Park, Wyoming. American Midland Naturalist, 133, 36–43.

[ece34688-bib-0025] Gralka, M. , Stiewe, F. , Farrell, F. , Möbius, W. , Waclaw, B. , & Hallatschek, O. (2016). Allele surfing promotes microbial adaptation from standing variation. Ecology Letters, 19, 889–898. 10.1111/ele.12625. 27307400 PMC4942372

[ece34688-bib-0026] Greenbaum, G. , Templeton, A. R. , Zarmi, Y. , & Bar‐David, S. (2014). Allelic richness following population founding events ‐ A stochastic modeling framework incorporating gene flow and genetic drift. PLoS One, 9(12), 1–23. 10.1371/journal.pone.0115203. PMC4272294 25526062

[ece34688-bib-0027] Günther, T. , & Coop, G. (2013). Robust identification of local adaptation from allele frequencies. Genetics, 195(1), 205–220. 10.1534/genetics.113.152462. 23821598 PMC3761302

[ece34688-bib-0028] Hagen, S. B. , Kopatz, A. , Aspi, J. , Kojola, I. , & Eiken, H. G. (2015). Evidence of rapid change in genetic structure and diversity during range expansion in a recovering large terrestrial carnivore. Proceedings of the Royal Society B: Biological Sciences, 282(1807), 20150092 10.1098/rspb.2015.0092. PMC4424642 25904665

[ece34688-bib-0029] Heppenheimer, E. , Cosio, D. S. , Brzeski, K. E. , Caudill, D. , Van Why, K. , Chamberlain, M. J. , … vonHoldt, B. (2018). Demographic history influences spatial patterns of genetic diversityin recently expanded coyote (*Canis latrans*) populations. Heredity, 120(3), 183–195. 10.1038/s41437-017-0014-5. 29269931 PMC5836586

[ece34688-bib-0030] Hill, J. K. , Collingham, Y. C. , Thomas, C. D. , Blakeley, D. S. , Fox, R. , Moss, D. , & Huntley, B. (2001). Impacts of landscape structure on butterfly range expansion. Ecology Letters, 4(4), 313–321. 10.1046/j.1461-0248.2001.00222.x.

[ece34688-bib-0031] Hinton, J. W. , Gittleman, J. L. , van Manen, F. T. , & Chamberlain, M. J. (2018). Size‐assortative choice and mate availability influences hybridization between red wolves (*Canis rufus*) and coyotes (*Canis latrans*). Ecology and Evolution, 8, 3927–3940. 10.1002/ece3.3950. 29721269 PMC5916303

[ece34688-bib-0032] Hody, J. W. , & Kays, R. (2018). Mapping the expansion of coyotes (*Canis latrans*) across America. ZooKeys, 97, 81–97. 10.3897/zookeys.759.15149. PMC5974007 29861647

[ece34688-bib-0033] Hofer, T. , Ray, N. , Wegmann, D. , & Excoffier, L. (2009). Large allele frequency differences between human continental groups are more likely to have occurred by drift during range expansions than by selection. Annals of Human Genetics, 73(1), 95–108. 10.1111/j.1469-1809.2008.00489.x. 19040659

[ece34688-bib-0034] Hughes, C. L. , Dytham, C. , & Hill, J. K. (2007). Modelling and analysing evolution of dispersal in populations at expanding range boundaries. Ecological Entomology, 32(5), 437–445. 10.1111/j.1365-2311.2007.00890.x.

[ece34688-bib-0035] Ibrahim, K. M. , Nichols, R. A. , & Hewitt, G. M. (1996). Spatial patterns of genetic variation generated by different forms of dispersal during range expansion. Heredity, 77, 282–291. 10.1038/hdy.1996.142.

[ece34688-bib-0036] Kabitzke, P. A. , Brunner, D. , He, D. , Fazio, P. A. , Cox, K. , Sutphen, J. , … Clayton, A. L. (2017). Comprehensive analysis of two Shank3 and the Cacna1c mouse models of autism spectrum disorder. Genes, Brain and Behavior, 17, 4–22. 10.1111/gbb.12405. 28753255

[ece34688-bib-0037] Kays, R. , Curtis, A. , & Kirchman, J. J. (2010). Rapid adaptive evolution of northeastern coyotes via hybridization with wolves. Biology Letters, 6(1), 89–93. 10.1098/rsbl.2009.0575. 19776058 PMC2817252

[ece34688-bib-0038] Kilgo, J. C. , Ray, H. S. , Ruth, C. , & Miller, K. V. (2010). Can coyotes affect deer populations in southeastern North America? The Journal of Wildlife Management, 74(5), 929–933. 10.2193/2009-263

[ece34688-bib-0039] Klopfstein, S. , Currat, M. , & Excoffier, L. (2006). The fate of mutations surfing on the wave of a range expansion. Molecular Biology and Evolution, 23(3), 482–490. 10.1093/molbev/msj057. 16280540

[ece34688-bib-0040] Li, H. , Handsaker, B. , Wysoker, A. , Fennell, T. , Ruan, J. , Homer, N. , Marth, G. , Abecasis, G. , & Durbin, R. (2009). The Sequence alignment/map format and SAMtools. Bioinformatics, 25(16), 2078–2079. 10.1093/bioinformatics/btp352. 19505943 PMC2723002

[ece34688-bib-0041] Lindblad‐Toh, K. , Wade, C. M. , Mikkelsen, T. S. , Karlsson, E. K. , Jaffe, D. B. , Kamal, M. , ... Lander, E. S. (2005). Genome sequence, comparative analysis and haplotype structure of the domestic dog. Nature, 438(7069), 803–819. 10.1038/nature04338. 16341006

[ece34688-bib-0042] Lischer, H. E. L. , & Excoffier, L. (2012). PGDSpider: An automated data conversion tool for connecting population genetics and genomics programs. Bioinformatics, 28(2), 298–299. 10.1093/bioinformatics/btr642. 22110245

[ece34688-bib-0043] Livezey, K. B. (2009). Range expansion of barred owls, parts I and II. The American Midland Naturalist, 161(1), 49–56. 10.1674/0003-0031-161.2.323.

[ece34688-bib-0044] Lotterhos, K. E. , & Whitlock, M. C. (2014). Evaluation of demographic history and neutral parameterization on the performance of FST outlier tests. Molecular Ecology, 23(9), 2178–2192. 10.1111/mec.12725. 24655127 PMC4228763

[ece34688-bib-0045] Lotterhos, K. E. , & Whitlock, M. C. (2015). The relative power of genome scans to detect local adaptation depends on sampling design and statistical method. Molecular Ecology, 24(5), 1031–1046. 10.1111/mec.13100. 25648189

[ece34688-bib-0046] Lunter, G. , & Goodson, M. (2011). Stampy: A statistical algorithm for sensitive and fast mapping of Illumina sequence reads. Genome Research, 21(6), 936–939. 10.1101/gr.111120.110. 20980556 PMC3106326

[ece34688-bib-0047] Luu, K. , Bazin, E. , & Blum, M. G. B. (2017). *pcadapt* : An R package to perform genome scans for selection based on principal component analysis. Molecular Ecology Resources, 17(1), 67–77. 10.1111/1755-0998.12592. 27601374

[ece34688-bib-0048] Mastro, L. L. (2011). Life history and ecology of coyotes in the Mid‐Atlantic states: A summary of the scientific literature. Southeastern Naturalist, 10(4), 721–730. 10.1656/058.010.0411

[ece34688-bib-0049] Mayr, E. (1954). Change of genetic environment and evolution In Huxley J., Hardy A. C., & Ford E. B. (Eds.), Evolution as a process (pp. 157–180). London, UK: Allen & Unwin.

[ece34688-bib-0050] McLaren, W. , Gil, L. , Hunt, S. E. , Riat, H. S. , Ritchie, G. R. S. , Thormann, A. , … Cunningham, F. (2016). The Ensembl variant effect predictor. Genome Biology, 17(1), 1–14. 10.1186/s13059-016-0974-4. 27268795 PMC4893825

[ece34688-bib-0051] Monzõn, J. , Kays, R. , & Dykhuizen, D. E. (2014). Assessment of coyote‐wolf‐dog admixture using ancestry‐informative diagnostic SNPs. Molecular Ecology, 23(1), 182–197. 10.1111/mec.12570. 24148003 PMC3899836

[ece34688-bib-0052] Nei, M. , Maruyama, T. , & Chakraborty, R. (1975). The Bottleneck effect and genetic variability in populations. Evolution, 29(1), 1–10. 10.1111/j.1558-5646.1975.tb00807.x 28563291

[ece34688-bib-0053] Norén, K. , Statham, M. J. , Ågren, E. O. , Isomursu, M. , Flagstad, Ø. , Eide, N. E. , … Sacks, B. N. (2015). Genetic footprints reveal geographic patterns of expansion in Fennoscandian red foxes. Global Change Biology, 21(9), 3299–3312. 10.1111/gcb.12922. 26058388

[ece34688-bib-0054] Nowak, R. M. (1979). North American Quaternary Canis. Lawrence, KS: Museum of Natural History, University of Kansas 10.5962/bhl.title.4072.

[ece34688-bib-0055] Nowak, R. M. (2002). The original status of wolves in eastern North America. Southeastern Naturalist, 1(2), 95–130. 10.1656/1528-7092(2002)001[0095:TOSOWI]2.0.CO;2

[ece34688-bib-0056] Omernik, J. M. , & Griffith, G. E. (2014). Ecoregions of the conterminous United States: Evolution of a hierarchical spatial framework. Environmental Management, 54, 1249–1266. 25223620 10.1007/s00267-014-0364-1

[ece34688-bib-0057] Pateman, R. M. , Hill, J. K. , Roy, D. B. , Fox, R. , & Thomas, C. D. (2012). Temperature‐dependent alterations in host use drive rapid range expansion in a butterfly. Science, 336(6084), 1028–1030. 10.1126/science.1216980. 22628653

[ece34688-bib-0058] Phillips, B. L. , Brown, G. P. , Travis, J. M. J. , & Shine, R. (2008). Reid’s Paradox revisited: The evolution of dispersal kernels during range expansion. The American Naturalist, 172(S1), S34–S48. 10.1086/588255. 18554142

[ece34688-bib-0059] Pritchard, J. K. , Stephens, M. , & Donnelly, P. (2000). Inference of population structure using multilocus genotype data. Genetics, 155(2), 945–959. 10.1111/j.1471-8286.2007.01758.x. 10835412 PMC1461096

[ece34688-bib-0060] Purcell, S. , Neale, B. , Todd‐Brown, K. , Thomas, L. , Ferreira, M. A. R. , Bender, D. , … Sham, P. C. (2007). PLINK: A Tool set for whole‐genome association and population‐based linkage analyses. The American Journal of Human Genetics, 81(3), 559–575. 10.1086/519795. 17701901 PMC1950838

[ece34688-bib-0061] Robinson, K. F. , Diefenbach, D. R. , Fuller, A. K. , Hurst, J. E. , & Rosenberry, C. S. (2014). Can managers compensate for coyote predation of white‐tailed deer? The Journal of Wildlife Management, 78(4), 571–579. 10.1002/jwmg.693

[ece34688-bib-0062] Rousset, F. (1997). Genetic differentiation and estimation of gene flow from F‐Statistics under isolation by distance. Genetics, 145, 1219–1228. 10.1002/ajmg.c.30221. 9093870 PMC1207888

[ece34688-bib-0063] Rutledge, L. Y. , Garroway, C. J. , Loveless, K. M. , & Patterson, B. R. (2010). Genetic differentiation of eastern wolves in Algonquin Park despite bridging gene flow between coyotes and grey wolves. Heredity, 105(6), 520–531. 10.1038/hdy.2010.6. 20160760

[ece34688-bib-0064] Saastamoinen, M. , Bocedi, G. , Cote, J. , Legrand, D. , Guillaume, F. , Wheat, C. W. , … del Mar Delgado, M. (2017). Genetics of dispersal. Biological Reviews, 358, 574–599. 10.1111/brv.12356. PMC5811798 28776950

[ece34688-bib-0065] Seehausen, O. (2004). Hybridization and adaptive radiation. Trends in Ecology and Evolution, 19(4), 198–207. 16701254 10.1016/j.tree.2004.01.003

[ece34688-bib-0066] Streicher, J. W. , McEntee, J. P. , Drzich, L. C. , Card, D. C. , Schield, D. R. , Smart, U. , … Castoe, T. A. (2016). Genetic surfing, not allopatric divergence, explains spatial sorting of mitochondrial haplotypes in venomous coralsnakes. Evolution, 70(7), 1435–1449. 10.1111/evo.12967. 27251954

[ece34688-bib-0067] Swaegers, J. , Mergeay, J. , Geystelen, A. V. A. N. , & Therry, L. (2015). Neutral and adaptive genomic signatures of rapid poleward range expansion. Molecular Ecology, 24(24), 6163–6176. 10.1111/mec.13462. 26561985

[ece34688-bib-0068] Szpiech, Z. A. , Jakobsson, M. , & Rosenberg, N. A. (2008). ADZE: A rarefaction approach for counting alleles private to combinations of populations. Bioinformatics, 24(21), 2498–2504. 10.1093/bioinformatics/btn478. 18779233 PMC2732282

[ece34688-bib-0069] Taulman, J. F. , & Robbins, L. W. (1996). Recent range expansion and distributional limits of the nine‐banded armadillo (*Dasypus novemcinctus*) in the United States. Journal of Biogeography, 23(5), 635–648. 10.1111/j.1365-2699.1996.tb00024.x.

[ece34688-bib-0070] Thornton, D. H. , & Murray, D. L. (2014). Influence of hybridization on niche shifts in expanding coyote populations. Diversity and Distributions, 20(11), 1355–1364. 10.1111/ddi.12253.

[ece34688-bib-0071] Travis, J. M. J. , & Dytham, C. (2002). Dispersal evolution during invasions. Evolutionary Ecology Research, 4(8), 1119–1129.

[ece34688-bib-0072] vonHoldt, B. , Heppenheimer, E. , Petrenko, V. , Croonquist, P. , & Rutledge, L. Y. (2017). Ancestry‐specific methylation patterns in admixed offspring from an experimental coyote and gray Wolf cross. Journal of Heredity, 108(4), 341–348. 10.1093/jhered/esx004. 28182234

[ece34688-bib-0073] vonHoldt, B. M. , Kays, R. , Pollinger, J. P. , & Wayne, R. K. (2016). Admixture mapping identifies introgressed genomic regions in North American canids. Molecular Ecology, 25(11), 2443–2453. 10.1111/mec.13667. 27106273

[ece34688-bib-0074] vonHoldt, B. M. , Pollinger, J. P. , Earl, D. A. , Knowles, J. C. , Boyko, A. R. , Parker, H. , … Wayne, R. K. (2011). A genome‐wide perspective on the evolutionary history of enigmatic wolf‐like canids. Genome Research, 21(8), 1294–1305. 10.1101/gr.116301.110. 21566151 PMC3149496

[ece34688-bib-0075] Voss, N. , Eckstein, R. L. , & Durka, W. (2012). Range expansion of a selfing polyploid plant despite widespread genetic uniformity. Annals of Botany, 110(3), 585–593. 10.1093/aob/mcs117. 22730022 PMC3400446

[ece34688-bib-0076] Wang, J. , Duncan, D. , Shi, Z. , & Zhang, B. (2013). WEB‐based GEne SeT AnaLysis Toolkit (WebGestalt): Update 2013. Nucleic Acids Research, 41, 77–83. 10.1093/nar/gkt439. PMC3692109 23703215

[ece34688-bib-0077] Wheeldon, T. , Patterson, B. , & White, B. (2010). Colonization history and ancestry of northeastern coyotes. Biology Letters, 6(2), 246–247; author reply 248–249. 10.1098/rsbl.2009.0822. 20089539 PMC2865032

[ece34688-bib-0078] Wheeldon, T. J. , Rutledge, L. Y. , Patterson, B. R. , White, B. N. , & Wilson, P. J. (2013). Y‐chromosome evidence supports asymmetric dog introgression into eastern coyotes. Ecology and Evolution, 3(9), 3005–3020. 10.1002/ece3.693. 24101990 PMC3790547

[ece34688-bib-0079] White, T. A. , Perkins, S. E. , Heckel, G. , & Searle, J. B. (2013). Adaptive evolution during an ongoing range expansion: The invasive bank vole (*Myodes glareolus*) in Ireland. Molecular Ecology, 22(11), 2971–2985. 10.1111/mec.12343. 23701376

[ece34688-bib-0080] Wilson, P. J. , Rutledge, L. Y. , Wheeldon, T. J. , Patterson, B. R. , & White, B. N. (2012). Y‐chromosome evidence supports widespread signatures of three‐species canis hybridization in eastern North America. Ecology and Evolution, 2(9), 2325–2332. 10.1002/ece3.301. 23139890 PMC3488682

[ece34688-bib-0081] Young, S. P. , & Jackson, H. H. T. (1951). The clever coyote. Harrisburg, PA: The Stackpole Company.

[ece34688-bib-0082] Zerbino, D. R. , Achuthan, P. , Akanni, W. , Amode, M. R. , Barrell, D. , Bhai, J. , … Flicek, P. (2017). Ensembl 2018. Nucleic Acids Research, 46, 754–761. 10.1093/nar/gkx1098. PMC5753206 29155950

[ece34688-bib-0083] Zhang, B. , Kirov, S. , & Snoddy, J. (2005). WebGestalt: An integrated system for exploring gene sets in various biological contexts. Nucleic Acids Research, 33, 741–748. 10.1093/nar/gki475. PMC1160236 15980575

