# Supplementary material for: High genomic diversity and candidate genes under selection associated with range expansion in eastern coyote (Canis latrans) populations
Source: Ecol Evol. 2018 Dec 4;8(24):12641–55. doi: 10.1002/ece3.4688 (PMC6309008; doi:10.1002/ece3.4688)
Supplement: Supplementary file 1 [file ECE3-8-12641-s001.docx]

Table S1: Sampling Location and collection year for each sample.

Please see excel file: Table_S1.xlsx

Table S2: Sequencing read counts for each sample at every filtering stage prior to SNP calling.

Please see excel file: Table_S2.xlsx

Table S3**:** Average ancestry coefficients (Q) for each sampling location in the ADMIXTURE

analysis:

| **Sampling Location** | **Q_Historical_** | **Q_Northeast_** | **Q_Southeas_**_t_ |
| --- | --- | --- | --- |
| **Historical Range** | | | |
| AZ | 1.000 | 0.000 | 0.000 |
| CA | 1.000 | 0.000 | 0.000 |
| ID | 1.000 | 0.000 | 0.000 |
| MN | 0.530 | 0.207 | 0.263 |
| MO | 0.295 | 0.008 | 0.697 |
| NE | 0.804 | 0.006 | 0.190 |
| NM | 0.914 | 0.002 | 0.083 |
| NV | 1.000 | 0.000 | 0.000 |
| OK | 0.300 | 0.001 | 0.700 |
| SK | 0.935 | 0.009 | 0.056 |
| TX | 0.878 | 0.008 | 0.115 |
| WA | 0.988 | 0.002 | 0.000 |
| WY | 1.000 | 0.000 | 0.000 |
| **OVERALL** | **0.868** | **0.019** | **0.113** |
| **Northeast Expansion** | | | |
| ME | 0.000 | 1.000 | 0.000 |
| NB | 0.000 | 1.000 | 0.000 |
| NJ | 0.000 | 1.000 | 0.000 |
| NY | 0.000 | 1.000 | 0.000 |
| ON | 0.000 | 1.000 | 0.000 |
| PA | 0.001 | 0.865 | 0.134 |
| **OVERALL** | **0.000** | **0.943** | **0.065** |
| **Southeast Expansion** | | | |
| AL | 0.000 | 0.000 | 1.000 |
| FL | 0.000 | 0.000 | 1.000 |
| GA | 0.000 | 0.000 | 1.000 |
| KY | 0.005 | 0.066 | 0.929 |
| LA | 0.015 | 0.250 | 0.735 |
| NC | 0.004 | 0.078 | 0.917 |
| SC | 0.000 | 0.000 | 1.000 |
| TN | 0.000 | 0.000 | 1.000 |
| VA | 0.001 | 0.233 | 0.766 |
| **OVERALL** | **0.002** | **0.065** | **0.933** |

Table S4: Genomic diversity statistics for each sampling location at putatively neutral (intergenic and in HWE) and genic loci.

|  | **Putatively Neutral Loci** | | **Genic Loci** | |  |
| --- | --- | --- | --- | --- | --- |
| **Sampling Location** | **H_o_** | **H_e_** | **H_o_** | **H_e_** |  |
| **Historical Range** | | | | | |
| AZ | 0.0215 | 0.0223 | 0.0236 | 0.0265 |  |
| CA | 0.017 | 0.0194 | 0.0223 | 0.0273 |  |
| ID | 0.0165 | 0.0176 | 0.0198 | 0.0228 |  |
| MN | 0.027 | 0.0263 | 0.0312 | 0.0311 |  |
| MO | 0.0162 | 0.0177 | 0.0195 | 0.0223 |  |
| NE | 0.0210 | 0.0225 | 0.0235 | 0.0264 |  |
| NM | 0.0229 | 0.0231 | 0.0268 | 0.0282 |  |
| NV | 0.0201 | 0.0215 | 0.0235 | 0.0260 |  |
| OK | 0.0243 | 0.0232 | 0.0300 | 0.0291 |  |
| SK | 0.0202 | 0.0196 | 0.0227 | 0.0234 |  |
| TX | 0.0245 | 0.0188 | 0.0278 | 0.0226 |  |
| WA | 0.0207 | 0.021 | 0.0253 | 0.0266 |  |
| WY | 0.0178 | 0.0175 | 0.0205 | 0.0209 |  |
| **Average** | **0.0207** | **0.0208** | **0.0243** | **0.0256** |  |
| **Northeast Expansion** | | | | | |
| ME | 0.0178 | 0.021 | 0.0230 | 0.029 |  |
| NB | 0.0157 | 0.0161 | 0.0213 | 0.0231 |  |
| NJ | 0.0201 | 0.0171 | 0.0263 | 0.0232 |  |
| NY | 0.0190 | 0.0159 | 0.0257 | 0.0220 |  |
| ON | 0.0237 | 0.0242 | 0.0306 | 0.0326 |  |
| PA | 0.0189 | 0.0229 | 0.0230 | 0.0304 |  |
| **Average** | **0.0192** | **0.0195** | **0.0250** | **0.0267** |  |
| **Southeast Expansion** | | | | | |
| AL | 0.0261 | 0.0258 | 0.0311 | 0.0321 |  |
| FL | 0.0218 | 0.0245 | 0.0262 | 0.031 |  |
| GA | 0.0247 | 0.0252 | 0.0296 | 0.0314 |  |
| KY | 0.0243 | 0.0251 | 0.0291 | 0.0312 |  |
| LA | 0.0222 | 0.0222 | 0.0262 | 0.0274 |  |
| NC | 0.0239 | 0.0249 | 0.0294 | 0.0321 |  |
| SC | 0.0263 | 0.0255 | 0.0326 | 0.0327 |  |
| TN | 0.0240 | 0.0181 | 0.0298 | 0.0229 |  |
| VA | 0.0181 | 0.0205 | 0.0217 | 0.0267 |  |
| **Average** | **0.0235** | **0.0235** | **0.0284** | **0.0297** |  |

Table S5: Outlier SNPs putatively associated with range expansion identified by either Bayenv2 or PCadapt. (Abbreviations: Chromosome, Chr; Bayes Factor, BF; intergenic, inter).

Please see excel file: Table_S5.xlsx

**Table S6**: Biological Process GO annotations for twelve outlier SNPs consistently associated with range expansion in the Bayenv2 and PCadapt analyses. (Abbreviations: Chromosome, Chr).

| **Chr** | **Position** | **Gene** | **Biological Process Annotations** |
| --- | --- | --- | --- |
| 2 | 43210227 | *5S_rRNA* | None |
| 10 | 15795366 | *KCNC2* | voltage-gated potassium channel activity, regulation of ion transmembrane transport, protein homooligomerization, potassium ion transmembrane transport, voltage-gated potassium channel activity, potassium ion transmembrane transport |
| 11 | 53204490 | *PAX5* | negative regulation of transcription by RNA polymerase II, transcriptional activator activity, RNA polymerase II proximal promoter sequence-specific DNA binding, transcription by RNA polymerase II, aging, lateral ventricle development, cerebral cortex development, adult behavior, skeletal muscle cell differentiation, positive regulation of transcription by RNA polymerase II, embryonic cranial skeleton morphogenesis, negative regulation of histone H3-K9 methylation |
| 16 | 53466454 | *WDR17* | None |
| 17 | 23407156 | *ALK* | peptidyl-tyrosine phosphorylation, activation of MAPK activity, transmembrane receptor protein tyrosine kinase activity, hippocampus development, adult behavior, swimming behavior, regulation of cell proliferation, protein autophosphorylation, neuron development, regulation of dopamine receptor signaling pathway, response to environmental enrichment |
| 20 | 3062963 | *EFCC1* | None |
| 20 | 40628561 | *ATRIP* | DNA damage checkpoint |
| 27 | 44159565 | *CACNA1C* | immune system development, positive regulation of cytosolic calcium ion concentration, heart development, high voltage-gated calcium channel activity, regulation of ion transmembrane transport, embryonic forelimb morphogenesis, camera-type eye development, calcium ion transmembrane transport via high voltage-gated calcium channel, membrane depolarization during action potential, membrane depolarization during AV node cell action potential, voltage-gated calcium channel activity involved in AV node cell action potential, regulation of heart rate by cardiac conduction, regulation of ventricular cardiac muscle cell action potential, membrane depolarization during atrial cardiac muscle cell action potential, membrane depolarization during action potential, high voltage-gated calcium channel activity |
| 28 | 10746279 | *ZDHHC16* | eye development, cellular response to DNA damage stimulus, heart development, protein palmitoylation |
| 33 | 4379522 | *EPHA6* | protein tyrosine kinase activity, peptidyl-tyrosine phosphorylation |
| 34 | 11841558 | *AHRR* | negative regulation of transcription by RNA polymerase II, transcriptional repressor activity, RNA polymerase II transcription factor binding, response to xenobiotic stimulus |
| 35 | 23379157 | *CARMIL1* | cell migration, lamellipodium assembly, positive regulation of cell migration, ruffle organization, macropinocytosis, urate metabolic process, positive regulation of stress fiber assembly, barbed-end actin filament uncapping, actin filament network formation, positive regulation of substrate adhesion-dependent cell spreading, positive regulation of lamellipodium organization, negative regulation of barbed-end actin filament capping |

Figure S1: Histrogram of missing data per locus over all 22,935 SNPs in 394 coyote samples. Mean proportion of missing data per locus is given by the dashed line.

Figure S2: Histrogram of missing data per indiviual over all 22,935 SNPs in 394 coyote samples. Mean proportion of missing data is given by the dashed line. Insert: Missing data per indiviual within each of the three sampled regions.

Figure S3: Density plot for the depth of sequencing coverage across all three sampling regions.

Figure S4: Histogram of minor allele frequencies over 22,935 SNPs within each of the sampled regions.

Figure S5: Cross-validation error per number of clusters (K) in the ADMIXTURE analysis.
